# Inverted papilloma of the bladder: a very rare benign lesion with malignant implications - case report and comprehensive literature review

**DOI:** 10.1016/j.ijscr.2025.111374

**Published:** 2025-04-25

**Authors:** Salim Ouskri, Youssef Kadouri, Jihad Lakssir, Imad Boualaoui, Hachem El sayegh, Yassine Nouini

**Affiliations:** a Ibn Sina Hospital, Morocco; b Souss Massa Hospital, Morocco

## Abstract

**Introduction:**

Bladder inverted papilloma (IP) is a rare benign urothelial lesion, representing 1–2.2 % of all bladder tumors. Although benign, IP can coexist with urothelial carcinoma, necessitating careful long-term follow-up.

**Case presentation:**

A 63-year-old male, chronic smoker presented with macroscopic hematuria and irritative urinary symptoms. Imaging and cystoscopy revealed multiple bladder polyps. Complete transurethral resection was performed, and histopathological examination confirmed the diagnosis of inverted papilloma. Follow-up cystoscopy at three months showed no recurrence.

**Discussion:**

Inverted papilloma commonly affects older males, typically presenting with hematuria or obstructive symptoms. Diagnosis requires histopathological confirmation demonstrating endophytic proliferation of urothelium without atypia. The risk of synchronous or subsequent urothelial carcinoma, particularly in the upper urinary tract, emphasizes the need for regular cystoscopic surveillance.

**Conclusion:**

Despite its benign nature, IP demands careful monitoring due to potential malignant associations. Endoscopic resection remains the standard treatment, followed by routine cystoscopic surveillance.

## Highlights

• Inverted papilloma (IP) is a rare benign bladder lesion (1–2.2%).
• Potentially associated with urothelial carcinoma, requiring follow-up.
• Male, 63 years, smoker, presenting hematuria and irritative symptoms.
• Ultrasound showed bladder polyps; treated by complete endoscopic resection.
• Histology confirmed benign inverted papilloma; no recurrence at 3 months.
• IP commonly presents in elderly males with hematuria.
• Diagnosis via histology: inverted growth without atypia.
• Risk of associated urothelial carcinoma mandates long-term cystoscopic surveillance.
• IP requires complete endoscopic removal.
• Regular cystoscopic follow-up recommended due to carcinoma risk.

## 1 Introduction

Urothelial carcinoma represents the predominant histologic type of bladder malignancy. According to the 2004 World Health Organization (WHO) classification, urothelial tumors encompass a spectrum of lesions, including papilloma, papillary urothelial neoplasm of low malignant potential (PUNLMP), low-grade and high-grade urothelial carcinoma, and noninvasive urothelial neoplasms. Within the latter category, entities such as carcinoma in situ, urothelial dysplasia, nephrogenic adenoma, and inverted papilloma (IP) are recognized.

Inverted papilloma of the bladder is an uncommon benign tumor, accounting for 1-2.2 % of bladder neoplasms [1]. Characterized by its endophytic growth, IP is diagnosed via histopathological examination and managed primarily through endoscopic resection. Despite its benign nature, its potential association with urothelial carcinoma mandates ongoing follow-up [ [2], X1].

## 2 Case report

We report the case of a 63-year-old male, a chronic smoker with a 25 pack-year history, who presented with intermittent gross hematuria for one month and lower urinary tract irritative symptoms, including frequency, urgency, and dysuria, without flank pain, fever, or weight loss. His past medical history included hypertension controlled by lisinopril, with no prior urological surgeries or family history of bladder cancer. Physical examination revealed mild suprapubic tenderness, no palpable masses, and a benign prostate on digital rectal examination.

**Fig. 1:**
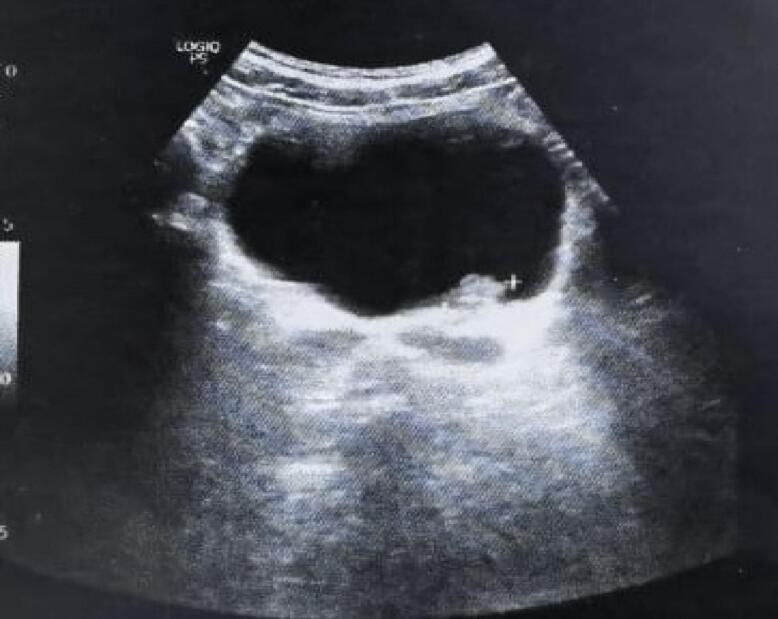
Ultrasound imaging of the bladder demonstrating intravesical polypoid masse.

Ultrasound examination identified two bladder polyps measuring 1.5 cm and 2.5 cm on the posterior wall and near the bladder neck, respectively, without dilation of the upper urinary tract Fig. 1. Urinary cytology was negative.

**Fig. 2:**
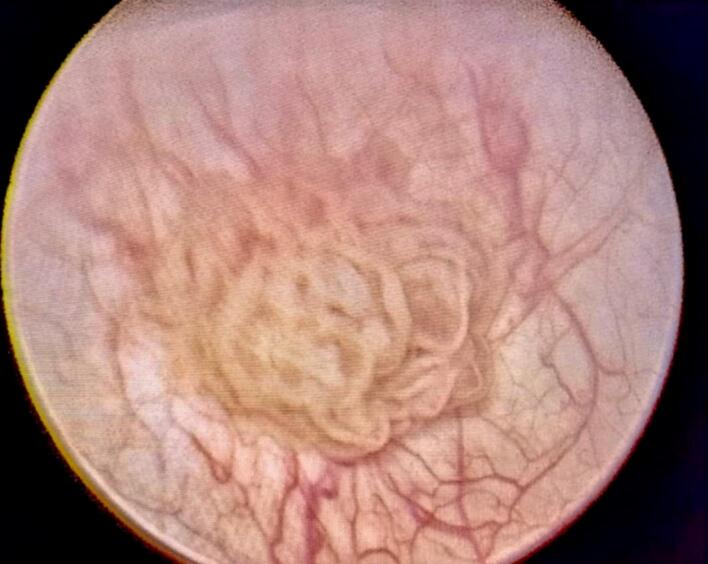
Cystoscopic view of the inverted papilloma.

Rigid cystoscopy under anesthesia confirmed the presence of multiple smooth polypoid lesions, including two larger lesions measuring approximately 2 cm and 3 cm in diameter. These were located on the posterior bladder wall and bladder neck. The remaining bladder mucosa appeared grossly normal, without signs of inflammation, trabeculation, or carcinoma in situ. Complete transurethral resection of the lesions was performed, including deep resection of the tumor bases down to the muscularis propria. Separate specimen containers were used to send the tumor fragments and deep muscle samples for pathological analysis (Fig. 2).

**Fig. 3:**
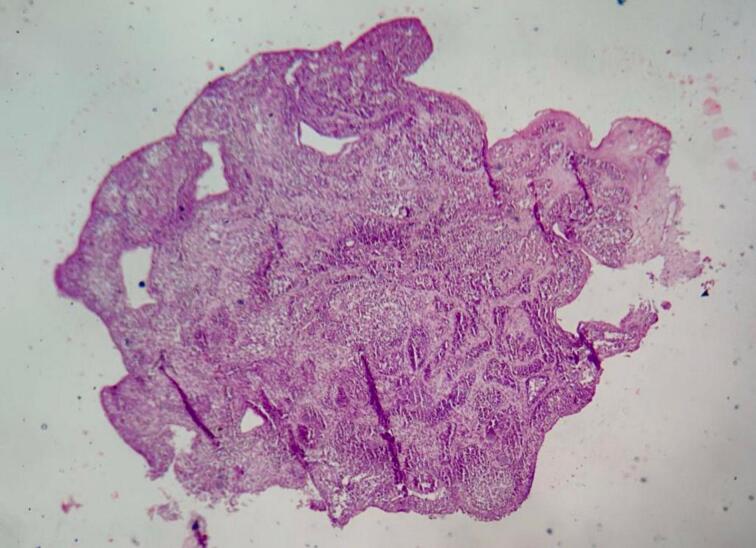
Histopathological examination (HE staining, ×20 magnification) demonstrates a benign urothelial proliferation characterized by endophytic (inverted) growth. The lesion is composed of anastomosing trabeculae and cords of histologically normal urothelial cells invaginating into the underlying lamina propria without evidence of cytological atypia, mitotic activity, or infiltration into the muscularis propria, consistent with a diagnosis of inverted papilloma.

**Fig. 4:**
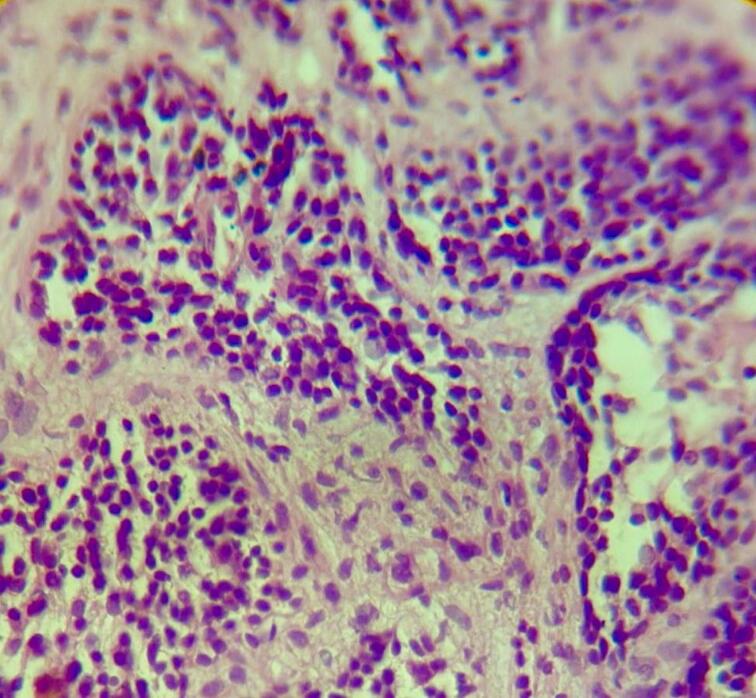
Higher magnification (HE staining, ×40) highlighting the cellular morphology of the inverted papilloma. The lesion is composed of medium-sized urothelial cells displaying regular, uniform nuclei without nuclear atypia or significant mitotic activity. Cells contain scant eosinophilic cytoplasm, arranged in cohesive cords and nests, confirming the benign nature of the inverted urothelial proliferation.

Histopathological analysis demonstrated an inverted growth pattern of uniform urothelial cells within the lamina propria, exhibiting minimal cytological atypia, rare mitotic figures, and eosinophilic microcysts. The overlying urothelium was intact, and immunohistochemistry showed low P53 expression and a Ki67 proliferation index of less than 5 %, confirming the benign nature of the lesion, diagnosed as inverted papilloma Fig. 3, Fig. 4.

At three-month follow-up, cystoscopy revealed no evidence of recurrence. Given the reported association with urothelial carcinoma, a surveillance plan was recommended: cystoscopy every six months for the first two years, followed by annual evaluations for at least five years.

## 3 Discussion

Inverted papilloma (IP) of the bladder is a rare urothelial lesion, first described by Paschkis in 1927 [3] and later named by Potts and Hirst in 1963 [4]. Representing approximately 1-2.2 % of bladder neoplasms [1], IP is distinguished by its inverted growth pattern. Although its etiology remains uncertain, some authors, including Cummings [5] and Matz et al. [6], have hypothesized that IP may represent a hyperplastic response to chronic inflammation or irritative stimuli rather than a true neoplastic process.

IP predominantly affects males in their sixth or seventh decade [7], with common presentations including hematuria or obstructive voiding symptoms. Ultrasonography typically reveals a solid bladder mass with a smooth or polypoid surface. Macroscopically, IP appears as a pedunculated or sessile polypoid lesion, usually less than 30 mm in diameter, with a predilection for the bladder neck and trigone [1].

Definitive diagnosis relies on histopathological examination, which demonstrates an endophytic proliferation of urothelial cells forming anastomosing islands and trabeculae that invaginate into the lamina propria without muscular invasion [1]. Henderson et al. [8] outlined diagnostic criteria, including normal transitional epithelium, uniform cytology without atypia or mitoses, and microcysts containing eosinophilic material. Kunze et al. [9] further classified IP into trabecular and glandular variants based on morphological distinctions.

Differential diagnosis is critical to distinguish IP from malignant urothelial neoplasms, such as papillary urothelial carcinoma or carcinoma in situ, which may also exhibit endophytic growth. Rarer differentials include nephrogenic adenoma and paraganglioma [10]. Accurate histopathological assessment is essential given IP’s benign prognosis and the need to rule out malignancy.

The standard treatment is complete endoscopic resection with coagulation of the tumor bed to minimize recurrence risk and ensure thorough histological evaluation. Historically, IP has been regarded as a benign lesion with a low recurrence rate [11]. However, recent literature highlights a significant association with urothelial carcinoma, necessitating long-term surveillance. A 2020 review by Smith et al. reported instances of malignant transformation and synchronous or metachronous urothelial carcinoma in IP patients, emphasizing the importance of vigilant follow-up.

A 2021 case series by Johnson et al. analyzed 50 IP cases, finding that 4 % had synchronous low-grade urothelial carcinoma, and 2 % developed metachronous carcinoma within five years. The authors recommended cystoscopic surveillance every six months for the first two years post-resection, followed by annual evaluations for at least five years. This aligns with earlier suggestions by Picozzi et al. [1], who proposed cystoscopy every four months initially, then every six months for three years, though more recent data support extended monitoring.

Molecular studies have provided additional clarity on IP’s nature. A 2019 study by Lee et al. revealed that IP exhibits fewer genetic alterations compared to urothelial carcinoma, lacking mutations in genes such as *TP53* and *FGFR3*, which are hallmarks of malignant urothelial tumors. This supports its classification as a benign entity but does not negate the need for clinical caution.

The association with urothelial carcinoma appears more pronounced in the upper urinary tract. Kimura et al. [12] reported a three-fold higher incidence of synchronous malignancy in ureteric IP (18 %) compared to bladder IP (6 %). Similarly, Cheng et al. [13] documented a 5.9 % rate of synchronous urothelial carcinoma in a series of 322 lower urinary tract IP cases. These findings underscore the importance of comprehensive evaluation and follow-up, particularly in atypical presentations or locations.

## 4 Conclusion

Inverted papilloma of the bladder is an uncommon, histopathologically diagnosed tumor managed effectively with endoscopic resection. Despite its benign classification, its association with urothelial carcinoma necessitates extended follow-up to monitor for recurrence or malignancy.

## 5 Methods

This the work has been reported in line with the SCARE criteria.

## CRediT authorship contribution statement

Salim Ouskri Urology Resident- IBN SINA HOSPITAL / salim.ouskri@gmail.com corresponding author.

Youssef kadouri Urologist- IBN SINA HOSPITAL / youcef.kadouri@gmail.com

Jihad Lakssir - Urology Assistant Professor, SOUSS MASSA HOSPITAL j.lakssir@gmail.com

Imad Boualaoui - Urology Assistant Professor, IBN SINA HOSPITAL

Email: imadboualaoui@gmail.com

Hachem El sayegh Urology Professor - IBN SINA HOSPITAL / hachemsayegh@yahoo.fr

Yassine Nouini Urology Professor- IBN SINA HOSPITAL / ynouini@yahoo.fr

## Consent

Written informed consent was obtained from the patient for publication and any accompanying images. A copy of the written consent is available for review by the Editor-in-Chief of this journal on request.

## Ethical approval

Ethical approval for this study was provided by the Ethical Committee of IBN SINA University Hospitals, Rabat, Morocco on 15/01/2025. Number of the decision is not delivered yet.

## Guarantor

Salim Ouskri

## Funding

NA.

## Registration of research studies

N/A

## Declaration of competing interest

NA.
